# Causal Evidence for the Role of Specific GABAergic Interneuron Types in Entorhinal Recruitment of Dentate Granule Cells

**DOI:** 10.1038/srep36885

**Published:** 2016-11-10

**Authors:** Cheng-Ta Lee, Min-Hua Kao, Wen-Hsien Hou, Yu-Ting Wei, Chin-Lin Chen, Cheng-Chang Lien

**Affiliations:** 1 Institute of Neuroscience, National Yang-Ming University, 155, Section 2, Li-Nong Street, Taipei 112, Taiwan; 2 Institute of Brain Science, National Yang-Ming University, 155, Section 2, Li-Nong Street, Taipei 112, Taiwan; 3 Brain Research Center, National Yang-Ming University, Taipei 112, Taiwan; 4 Charité Universitätsmedizin Berlin, 10117 Berlin, Germany

## Abstract

The dentate gyrus (DG) is the primary gate of the hippocampus and controls information flow from the cortex to the hippocampus proper. To maintain normal function, granule cells (GCs), the principal neurons in the DG, receive fine-tuned inhibition from local-circuit GABAergic inhibitory interneurons (INs). Abnormalities of GABAergic circuits in the DG are associated with several brain disorders, including epilepsy, autism, schizophrenia, and Alzheimer disease. Therefore, understanding the network mechanisms of inhibitory control of GCs is of functional and pathophysiological importance. GABAergic inhibitory INs are heterogeneous, but it is unclear how individual subtypes contribute to GC activity. Using cell-type-specific optogenetic perturbation, we investigated whether and how two major IN populations defined by parvalbumin (PV) and somatostatin (SST) expression, regulate GC input transformations. We showed that PV-expressing (PV+) INs, and not SST-expressing (SST+) INs, primarily suppress GC responses to single cortical stimulation. In addition, these two IN classes differentially regulate GC responses to θ and γ frequency inputs from the cortex. Notably, PV+ INs specifically control the onset of the spike series, whereas SST+ INs preferentially regulate the later spikes in the series. Together, PV+ and SST+ GABAergic INs engage differentially in GC input-output transformations in response to various activity patterns.

The hippocampus is a key brain structure for cognitive and emotional functions^1^,^2^,^3^,^4^. Among hippocampal subregions, the dentate gyrus (DG) is the first station and serves as the gatekeeper of the hippocampus^5^,^6^,^7^,^8^. Granule cells (GCs), the glutamatergic projection neurons, constitute the vast majority of the cell population in the DG and are essential for memory formation and retrieval and for complex information processing in the hippocampal network^6^,^9^,^10^,^11^. Under physiological conditions, only a small fraction (1–2%) of the GC population can be activated simultaneously by a barrage of excitatory inputs from the entorhinal cortex (EC) and covey cortical signals to the downstream CA3 region^12^,^13^,^14^. This phenomenon has been referred to as “sparse population coding”, which is thought to be essential for information encoding and pattern separation^15^. Several factors may constrain the number of active GCs in response to synaptic inputs. First, GCs have particularly negative membrane potential and relatively low input resistance^16^,^17^,^18^. Second, the GC dendrites are passive and leaky, thereby strongly attenuating synaptic inputs along the dendrites^18^,^19^. Lastly, GCs receive strong local inhibition originating from local-circuit GABAergic interneurons (INs)^20^,^21^,^22^,^23^.

Inhibition within the DG circuitry has been identified as a prime mediator of the sparse activation of GCs^5^,^24^,^25^,^26^,^27^. A diverse population of GABAergic INs with distinct functions is known to exist in the hippocampus^28^,^29^,^30^. For example, parvalbumin-expressing (PV+) inhibitory cells control the action potential initiation of principal neurons via axonal innervations onto perisomatic areas of principal neurons^20^,^31^,^32^. In contrast, somatostatin-expressing (SST+) inhibitory cells, a major type of dendrite-targeting GABAergic cells, regulate dendritic Na^+^ or Ca^2+^ spikes and synaptic plasticity by innervating dendritic domains of principal neurons^32^,^33^. Domain-specific GABAergic INs in the hippocampus also constitute two recurrent inhibitory circuits with distinct operating modes^34^. Perisomatic inhibitory INs are time-locked to the onset of the action potential series and transiently inhibit the somatic and perisomatic regions of pyramidal cells (PCs). In contrast, dendrite-targeting INs are activated in proportion to the rate of action potentials in the series and durably inhibit the distal apical dendrites^34^. However, the causal roles of specific GABAergic IN types involved in the processing of information passing from the EC to the hippocampus have not been established.

In this study, we investigated the inhibitory control of GC input-output (I–O) transformations by PV+ and SST+ INs, the two major IN populations in the DG circuit *in vitro*. Using cell-type-specific optogenetic perturbation, we found that GC responses to single entorhinal stimulation were controlled by PV+ INs but not by SST+ INs. However, both IN classes differentially regulated spiking activity evoked by theta-gamma frequency stimulations. Through distinct inhibitory mechanisms, the DG can generate distinct spike outputs during different states of cortical activity.

## Results

### GABAergic mechanism gates entorhinal activation of GCs

**Figure 1:**
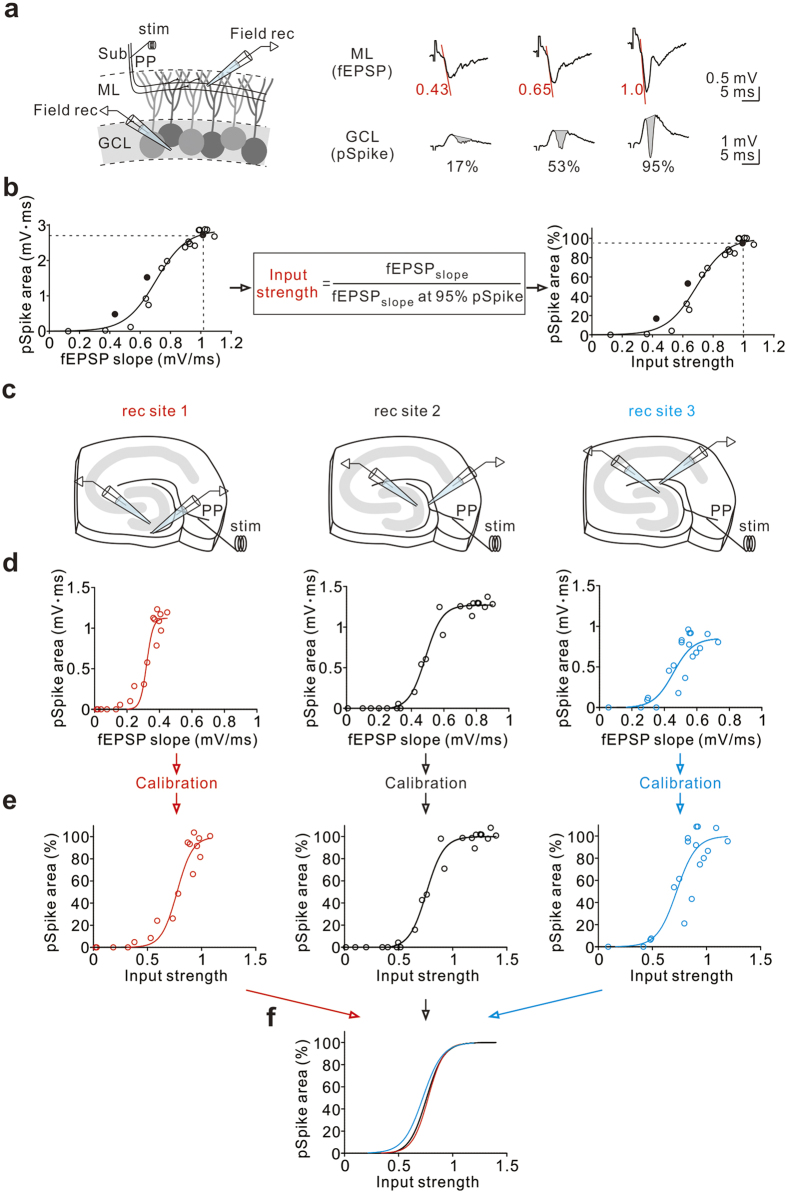
Calibration of input strength. (**a**) Left, schematic of recording configuration. A stimulation electrode (stim) was placed in the subiculum (Sub) to activate the PP fibres; two field-recording (Field rec) electrodes were placed, one in the GCL and one in the ML, to simultaneously detect the pSpike and fEPSP, respectively, in response to single pulses delivered to the PP at varying stimulus strengths. Right, example traces of fEPSP and pSpike recordings in response to PP stimulation. The fEPSP slope (red lines, absolute values of slope) was calculated as the slope of the rising phase at 20 to 50% of the peak response. The pSpike was calculated based on the area (in grey). (**b**) Left, plot of pSpike area against fEPSP slope for the experiment illustrated in (**a**). Middle, the definition of input strength; input strength is defined as the slope of the fEPSP (fEPSP_slope_, red lines) elicited at any given stimulus intensity normalized to the fEPSP_slope_ evoked at a stimulus intensity that results in a pSpike of 95% of its maximal amplitude. Right, normalized pSpike area plotted against input strength. Data are fitted with a sigmoid function. Filled symbols correspond to the example traces in (**a**). Dashed lines indicate the 95% pSpike and the corresponding fEPSP slope (left) or input strength (right). (**c**) Schematic of recording at three different locations. After converting to input strength, the two recording electrodes (left, at rec site #1) were simultaneously moved to the other recording sites (middle, site #2 and right, site #3) for comparing the effects of calibrations. (**d**) The pSpike area is plotted against fEPSP slope for the experiment illustrated in (**c**) at three indicated recording sites. Data are fitted with a sigmoid function. (**e**) The normalized pSpike area is plotted against input strength for the same experiments as in (**d**). Data are fitted with a sigmoid function. (**f**) The fitting curves in (**e**) at three different recording sites are merged together in the same plot.

Activity of individual GCs in response to glutamatergic inputs is primarily restrained by powerful GABAergic inhibition^26^,^35^,^36^,^37^. We used extracellular recording techniques to examine the influence of GABAergic inhibition on the GC responses to the cortical input, the perforant path (PP). As described previously^36^,^38^, we first calibrated the input strength in individual rodent brain slices in the presence of two field-recording electrodes: one electrode placed in the molecular layer (ML), for field excitatory postsynaptic potentials (fEPSPs), and one in the GC layer (GCL), for population spikes (pSpikes) (Fig. 1a). The GC population response was quantified as the pSpike area (Fig. 1a; see also ref. 39). The pSpike area increased sigmoidally as a function of the fEPSP slope (Fig. 1b left). Accordingly, the fEPSP slope evoked at any given stimulus intensity was normalized to the fEPSP slope elicited by a stimulus intensity that resulted in a pSpike area at 95% of its maximum (see also the equation in the box; Fig. 1b middle). The normalized fEPSP slope in this study is referred to as the input strength (Fig. 1b right). Although the magnitudes of both fEPSPs and pSpikes were highly location-specific, the GC population response-input strength relation was independent of recording sites within the same slice (Fig. 1c–f). This advantage allowed us to average the data from different slices^36^.

**Figure 2:**
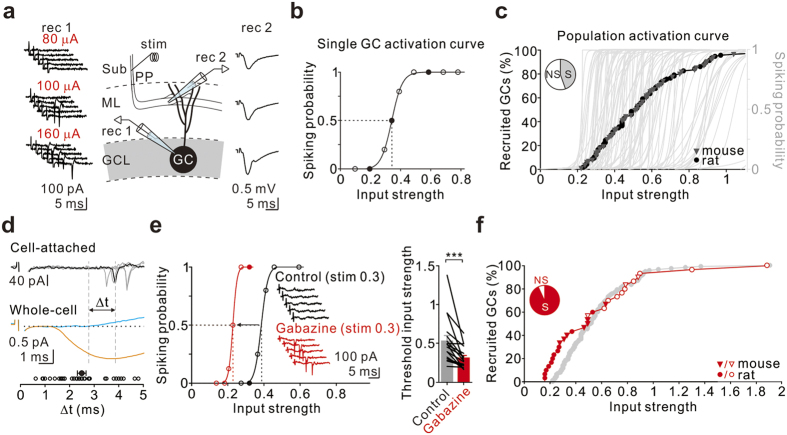
GABA_A_ receptor conductance regulates GC I-O transformations. (**a**) Schematic of recording configuration: example traces of action currents (left, rec 1 represents loose-patch recordings) recorded from a single GC and fEPSP recordings (right, rec 2) in response to PP stimulation. (**b**) Plot of spiking probability against input strength for a GC (sigmoidal fit, dashed lines indicate the threshold input strength). Filled symbols correspond to the example traces. (**c**) Activation curve of the GC population (cumulative plot of threshold input strengths, n = 115/259). Filled circles and grey triangles correspond to GCs from rats and mice, respectively. Grey sigmoids, the activation curves of individual GCs as plotted in (**b**). Pie chart, the percentages of spiking (S) and non-spiking (NS) GCs under PP stimulation. (**d**) Top traces represent cell-attached recordings from a single GC in response to threshold PP stimulation (5 superimposed sweeps). Bottom traces represent voltage-clamp recordings from the same neuron; the EPSC (orange, average of 10 sweeps) was recorded at −75 mV and the concomitant IPSC (blue, isolated by subtraction from average of 10 sweeps) was recorded at −55 mV. The vertical dashed line on the left indicates the onset of the IPSC and the right one indicates the average spike timing of the example GC. Bottom, summary of 37 experiments. Filled symbol represents the mean Δt. (**e**) Spiking probability for one GC plotted against input strength before (black) and after (red) gabazine treatment (sigmoidal fit). Inset, loose-patch recording at 0.3 input strength before (black) and after (red) gabazine treatment. Filled symbols correspond to the example traces. Bar graph, gabazine treatment decreased the threshold input strength of spiking GCs. Grey and red bars showing the average threshold input strength from GCs (n = 17) before and after gabazine treatment, respectively. Error bars indicate the SEM. ***P < 0.001. (**f**) Population activation curves after gabazine treatment (n = 30). Filled and open symbols correspond to spiking and non-spiking GCs, respectively, before gabazine treatment. Pie chart showing the percentages of spiking (S) and non-spiking (NS) GCs after gabazine treatment. Grey symbols correspond to GCs without gabazine treatment as shown in (**c**).

We next established the activation curve of single GCs in response to cortical stimulation. To achieve this, we simultaneously recorded action currents (“spikes”) in the cell-attached configuration from individual GCs along with the corresponding fEPSPs over a range of stimulus intensities (Fig. 2a). The spiking probability was plotted against the input strength and fitted with a sigmoid function. The input strength that evoked a 50% spike probability in the recorded cells was defined as the threshold input strength (Fig. 2b). In agreement with previous studies^12^,^35^, 44% of recorded GCs (62 of 131 from rats; 53 of 128 from mice) were recruited (“spiking GCs”) with this stimulation paradigm (Fig. 2c, “S” in the pie chart). Because similar results were obtained from rats and mice, all data were pooled (Fig. 2c). Finally, a cumulative distribution of threshold input strengths from all spiking GCs (rats and mice) was used to build the population activation curve, which represents the fractional recruitment of the GC population as a function of the input strength (n = 115 cells; Fig. 2c).

Inhibition is known to shape the neuronal I-O relationship^26^,^35^,^40^,^41^. We noticed that GCs, unlike CA1 PCs, were insensitive to input strengths of less than 0.2 (Fig. 2c). Does inhibition (whether hyperpolarizing or shunting) contribute to the offset of the I-O relationship? To answer this question, we first compared the timing of the spike elicited in GCs by PP stimulation with the onset of the inhibitory postsynaptic current (IPSC). When stimulated at the threshold for spike generation, the spike occurred 2.49 ± 0.18 ms (Δt) after the onset of the IPSC (n = 37; Fig. 2d). Notably, the excitatory postsynaptic current (EPSC) occurred before the IPSC (Fig. 2d); on average, the latency between the EPSC and IPSC was 3.17 ± 0.22 ms (n = 37). This temporal sequence suggests that stimulating the PP in the subiculum evoked disynaptic inhibition in GCs and further confirms a lack of contribution of GABAergic conductance to the fEPSP slope. Thus, PP-induced synaptic inhibition impinged on GCs before the membrane potential of the neuron reached the threshold for spike generation, thereby offsetting the I-O relationship. Consistent with this idea, removing GABAergic inhibition by using the GABA_A_ receptor antagonist gabazine (1 μM) increased the spiking probability of individual GCs with the same input strength (Fig. 2e left), leading to a significant reduction in the threshold input strength required to activate GCs (control, 0.53 ± 0.07; gabazine, 0.31 ± 0.03, n = 17; P = 0.0003, Wilcoxon signed-rank test; Fig. 2e right). In addition to a reduction of the threshold input strength, gabazine also transformed most non-spiking GCs into spiking GCs (Fig. 2f). By decreasing the threshold input strength of spiking GCs and recruiting some non-spiking GCs, gabazine greatly increased the fraction of active neurons in response to the cortical input (control, 44%; gabazine, 91%; Fig. 2f). Our results demonstrate that GABAergic inhibition sets a high input threshold for GCs, thereby ensuring sparse activity in the DG.

### Specific IN subtypes regulate GC I-O transformations

**Figure 3:**
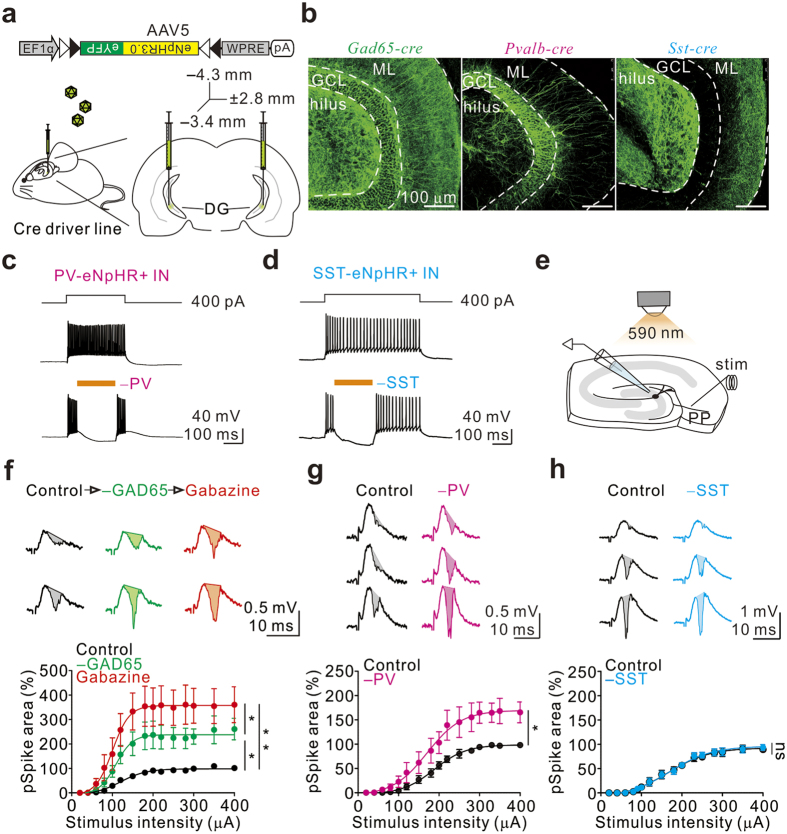
PV+ INs, but not SST+ INs, regulate GC responses to single-shock stimulation of PP. (**a**) Schematic of a mouse brain injected with an AAV5 vector carrying EF1α-DIO-eNpHR3.0-eYFP into the ventral hippocampal DG. (**b**) Left to right, two-photon image stacks of the ventral DG from *Gad65*-, *Pvalb*-, and *Sst-cre* mice 6 weeks after virus injection. (**c**) Example spikes evoked by current pulse injection (top) in a PV-eNpHR+ IN in the absence (middle) and in the presence of optogenetic silencing (bottom). (**d**) The same experiment as (**c**) in a SST-eNpHR+ IN. (**e**) Schematic of the recording configuration for (**f**–**h**). (**f**) Top, example traces of pSpike recordings from the GCL under control conditions (black, left), under light stimulation (−GAD65; green, middle), and after gabazine treatment (red, right) evoked by PP stimulation at two different stimulus intensities in a *Gad65-cre* mouse. Bottom, pSpike area is plotted against stimulus intensity under control conditions (black), light stimulation (green), and after gabazine treatment (red) in *Gad65*-*cre* mice (sigmoidal fit to the data-points). Error bars indicate the SEM. *P < 0.05, **P < 0.01. (**g**) The same experimental configuration as in (**f**) was used for *Pvalb-cre* mice. *P < 0.05. (**h**) The same experimental configuration as in (**f**) was used for *Sst-cre* mice. ns, not significant.

GABAergic inhibitory INs are heterogeneous, and individual subtypes serve distinct inhibitory roles in network activity^25^,^37^,^42^,^43^,^44^,^45^. They can be divided into two main classes according to the target selectivity of the axon: somatic INs versus dendritic INs^22^,^37^,^38^,^46^,^47^. PV+ INs, mainly basket cell (BC)-like cells (i.e., BCs and axo-axonic cells), constitute soma-targeting INs^48^,^49^, whereas SST+ INs, a large fraction of which are hilus perforant path associated (HIPP)-like cells, are a major subpopulation of dendrite-targeting INs in the DG^45^. Notably, SST+ dendrite-targeting INs, but not PV+ soma-targeting INs, are known to increase the firing rate of CA1 PCs in response to input from the Schaffer collateral^50^, but whether specific IN classes contribute to DG GC responses to the PP input has not been resolved. To address this question, we sought to silence specific types of GABAergic INs via optogenetic inhibition. As illustrated in Fig. 3a, we bilaterally injected an adeno-associated virus serotype 5 (AAV5) expressing Cre-dependent enhanced *Natronomonas* halorhodopsin-3.0 (eNpHR3.0)-eYFP [AAV5:(*eNpHR-eYFP*)^Cre^] into the ventral DG of Glutamic acid decarboxylase 65 (*Gad65*)-, *Pvalb*-, and *Sst-cre* mice. Consistent with the presence of Cre expression in GAD65+ neurons, eYFP signals were detected across all laminated areas in the DG in slices from *Gad65-cre* mice (Fig. 3b left). In contrast, strong eYFP signals in slices from *Pvalb-cre* mice were exclusively detected in the GCL (Fig. 3b middle), whereas intense eYFP signals in the outer-third and the middle-third of the ML and in the hilus were observed in slices from *Sst-cre* mice (Fig. 3b right). The expression of eNpHR-eYFP was functional because whole-cell recordings from eYFP+ neurons showed that delivery of amber light rapidly terminated neuronal spiking and caused subsequent membrane hyperpolarization (Fig. 3c,d). We next assessed the influence of specific IN classes on GC population activity by measuring the pSpikes in the DG in response to single-shock PP stimulation (0.033 Hz; Fig. 3e–h). Silencing of GAD65+ INs greatly increased the pSpike tested at various stimulus intensities (maximal effect, 252 ± 43% of control, n = 7; P = 0.02, Wilcoxon signed-rank test; Fig. 3f). Interestingly, we noticed that the effect was weaker than that of gabazine application (n = 7; P = 0.03, Wilcoxon signed-rank test; Fig. 3f), suggesting that spontaneous and miniature IPSCs as well as tonic inhibition likely have a significant effect on the activation of GCs. Similarly, silencing of PV+ INs also increased the pSpike. However, the effect of silencing GAD65+ INs was ~2-fold greater than that of silencing PV+ INs (maximal effect, 175 ± 22% of control, n = 6; P = 0.03, Wilcoxon signed-rank test; Fig. 3g). In contrast, silencing of SST+ INs had no effect on the pSpike evoked by single-shock PP stimulation at any given intensities (maximal effect, 105 ± 3% of control, n = 5; P = 0.31, Wilcoxon signed-rank test; Fig. 3h). It is important to note that the lack of effect of SST+ cells was not a result of insufficient expression of eNpHR-eYFP or due to differential labelling of one cell type over another (Supplementary Fig. 1). Our results indicated that PV+ INs, but not SST+ INs, regulate GC I-O transformations in response to single-shock stimulation.

**Figure 4:**
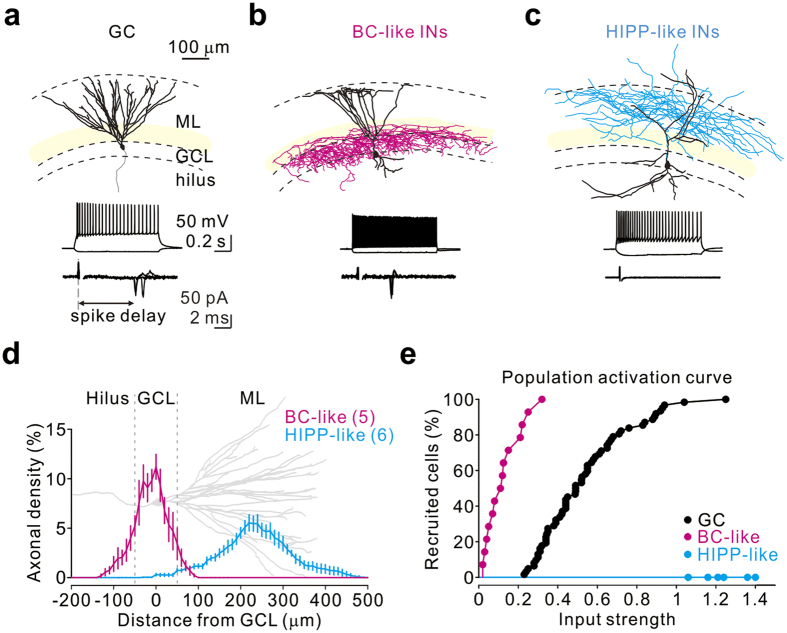
PP input preferentially recruits BC-like cells over HIPP-like cells. (**a**–**c**) Reconstruction (top), spiking pattern (middle), and cell-attached recording traces (bottom) from three representative cells. From left to right: (**a**) a GC, which displayed mature GC morphology (axon in grey); (**b**) a BC-like cell, which had characteristically dense axonal arborization (magenta) within the GCL; (**c**) a HIPP-like cell, which had the axonal distribution in the PP terminal field (cyan). Somata and dendrites are indicated in black. From the bottom to the top, dashed lines mark the margins of the hilus, GCL, and ML. BC-like cells exhibited a fast-spiking firing pattern; HIPP-like cells displayed non-fast spiking patterns. Cell-attached recordings show spikes, detected as action currents (5 superimposed sweeps), evoked at the threshold input strength of each neuron. Note that HIPP-like cells cannot be recruited by the maximal input strength. (**d**) Axonal density distribution for BC-like cells (magenta, n = 5) and HIPP-like cells (cyan, n = 6) plotted against distance from the GCL. A reconstructed GC (grey) is aligned and scaled to the plot for reference. (**e**) Population activation curves for GCs (black), BC-like cells (magenta), and HIPP-like cells (cyan).

To affect GC spiking, soma-targeting INs must discharge before GCs in response to PP stimulation and must have a relatively low threshold input strength. Here, we examined the spiking of INs in response to threshold PP stimulation (i.e., 0.5 input strength) and compared their spike timing with GCs (Fig. 4a–c, Supplementary Fig. 2). We first performed a correlated analysis of the morphological properties of all recorded cells by using *post hoc* biocytin labelling (Fig. 4a–c, Supplementary Fig. 2). We next identified BC-like (including BCs and axo-axonic cells) and HIPP-like cells, the latter being the major population of SST+ cells, according to axonal target selectivity, somatic locations, and physiological properties^22^,^37^,^43^,^51^. In total, 14 cells, each of which displayed high-frequency action potentials (>120 Hz) and relatively low input resistance (101.67 ± 10.89 MΩ) at ~32 °C, were identified as putative BC-like cells. Among them, 6 cells showed intact axon staining (note that one cell with dense axonal puncta exclusively in the GCL was difficult to digitally reconstruct). With the GCL as reference (Fig. 4d), reconstructed BC-like cells showed the highest axonal density distribution within the GCL, whereas HIPP-like cells displayed the majority of axonal density distribution in the outer two-thirds of the ML. Strikingly, BC-like cells spiked earlier than GCs (2.94 ± 0.25 ms prior to GCs, n = 14; P < 0.0001, Wilcoxon rank-sum test; Supplementary Fig. 2h), whereas no HIPP-like cells (n = 6; Fig. 4c,e) were recruited by the maximal input stimulation (i.e., 1.0 input strength). Analysis of activation curves revealed that the majority of BC-like cells (n = 10/14) were recruited by PP stimulation below an input strength of 0.2, at which no GCs were recruited (n = 0/115; Fig. 4e). In contrast, no HIPP-like cells were recruited by any input strength up to 1.4 under the same conditions.

### PV+ INs set the input threshold and constrain the fraction of active GCs

**Figure 5:**
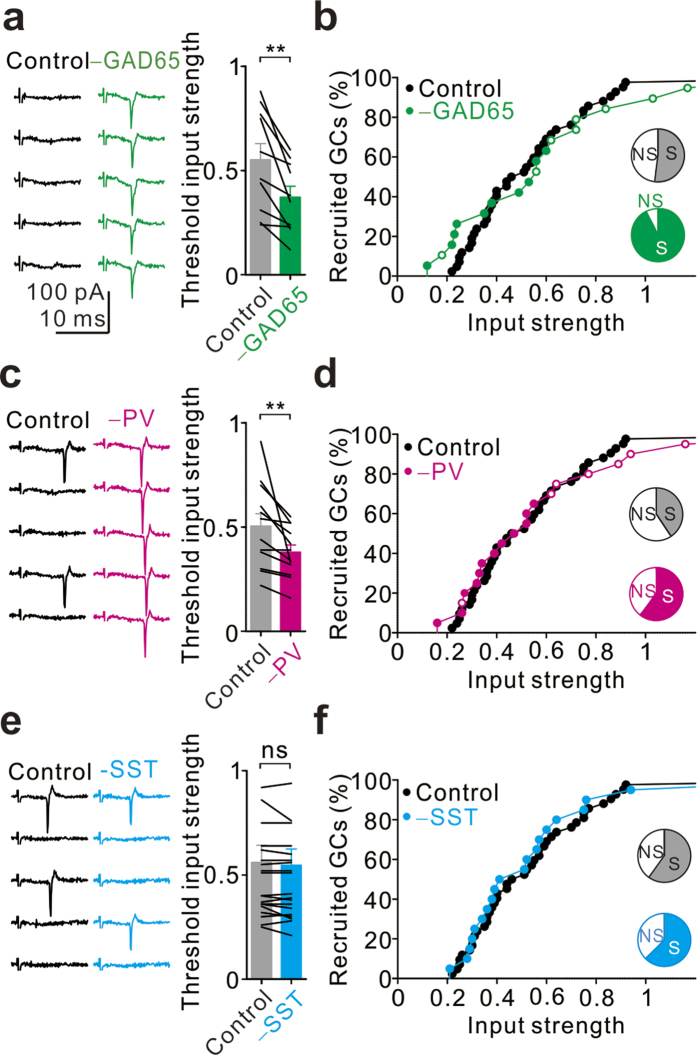
Silencing PV+ INs, but not SST+ INs, reduces the GC spiking threshold and increases the percentage of spiking GCs. (**a**) Cell-attached recordings from a single GC before (black, left) and during light stimulation (green, right) at threshold input strength in a *Gad65*-*cre* mouse. Summary plot of threshold input strengths from GCs (n = 10) without and with light stimulation in *Gad65*-*cre* mice. Error bars indicate the SEM. **P < 0.01. (**b**) Population activation curves under control conditions (black, n = 41) and under light stimulation (green, n = 19). (**c**) The same experiments as (**a**) in *Pvalb-cre* mice. (**d**) Population activation curves under control conditions (black, n = 41) and under light stimulation (magenta, n = 20). (**e**) The same experiments as (**a**) in *Sst-cre* mice. ns, not significant. (**f**) Population activation curves before (black, n = 41) and during light stimulation (cyan, n = 20).

We next investigated the effect of silencing genetically defined INs on the activation curves of individual GCs and the GC population. Similar to the GABA_A_ receptor blockade, optogenetic silencing of GAD65+ INs caused a dramatic increase in the spiking probability and a reduction in the threshold input strength of single GCs (n = 10; P = 0.002, Wilcoxon signed-rank test; Fig. 5a). In addition, silencing of GAD65+ INs activated most non-spiking GCs (Fig. 5b; pie chart; non-spiking (NS) percentage decreased from 48% to 7%). Likewise, optogenetic silencing of PV+ INs significantly increased the spiking probability and decreased the threshold input strength of individual GCs (n = 12; P = 0.004, Wilcoxon signed-rank test; Fig. 5c). Furthermore, it activated some non-spiking GCs (NS percentage decreased from 60% to 40%; pie chart in Fig. 5d). Conversely, neither the threshold input strength of individual GCs (n = 20; P = 1, Wilcoxon signed-rank test; Fig. 5e) nor the percentage of spiking GCs (60% in control *vs*. 63% in –SST; Fig. 5f) was affected by silencing of SST+ INs under the same conditions. As a result, optogenetic silencing of SST+ INs had a minimal influence on the activation curve of the GC population under conditions of sparse network activity (Fig. 5f). Together, these results indicate that PV+ INs rather than SST+ INs primarily regulate GC excitability in response to sparse cortical activity.

### PV+ and SST+ INs differentially entrain the firing of GCs during θ and γ frequency inputs

**Figure 6:**
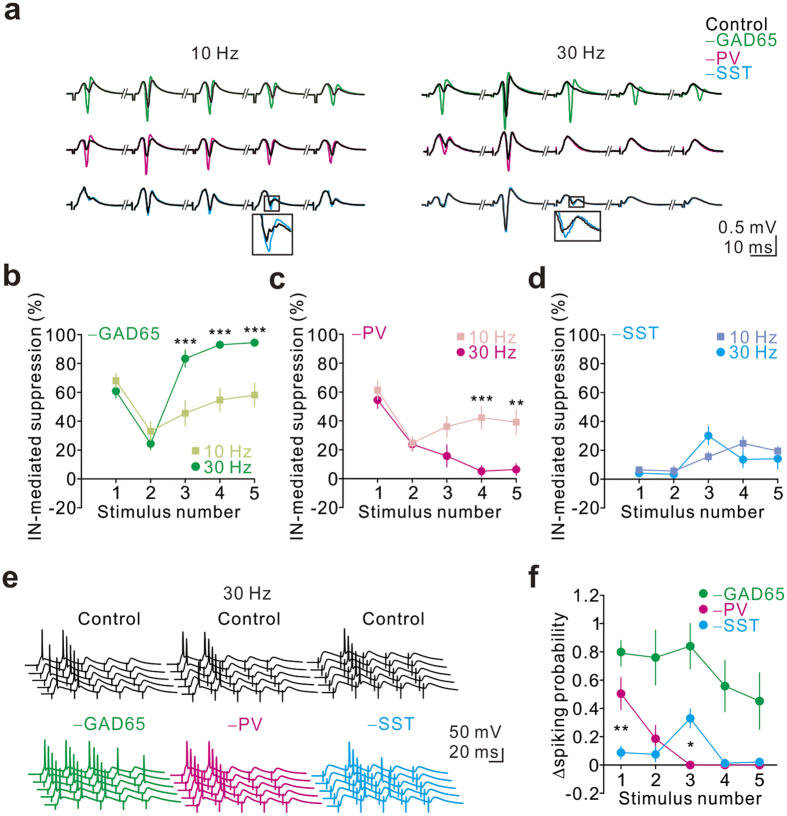
PV+ and SST+ INs differentially shape GC responses to repetitive synaptic input. (**a**) From the top to the bottom, pSpikes were evoked by PP stimulation at 10-Hz (left) and 30-Hz (right) trains before (black) and after silencing of GAD65+ (−GAD65, green), PV+ (−PV, magenta), and SST+ (−SST, cyan) INs. Plateau stimulation intensity was used to evoke maximal pSpikes. Amber light was presented during trains. The insets show the enlargement of the traces in the squares. (**b**)–(**d**) Summary plots of GAD65+, PV+, and SST+ IN-mediated suppression versus number of stimuli. The degree of IN-mediated suppression (%) was quantified as 100× (pSpike area-IN – pSpike area-Control)/pSpike area-IN. Two-way repeated-measures ANOVA for comparison of 10 Hz versus 30 Hz across multiple numbers of stimuli; *post hoc* Bonferroni’s test for testing the significance between 10 Hz and 30 Hz at each stimulus number. **P < 0.01, ***P < 0.001. (**e**) From left to right, whole-cell current-clamp recordings from GCs in response to PP stimulation at near-threshold strength before (top, black) and after optogenetic silencing of GAD65+ (green), PV+ (magenta) or SST+ (cyan) INs. (**f**) Summary plot of the change in spiking probability of GCs against the number of stimuli following silencing of GAD65+ (green), PV+ (magenta) or SST+ (cyan) INs. For PV+ *vs*. SST+, *P < 0.05, **P < 0.01.

In the engaged hippocampal network, the DG exhibits θ (4–8 Hz) and γ (30–80 Hz) frequency oscillations, both of which depend mainly on excitatory inputs from the EC^52^,^53^,^54^. With cell-type-specific targeting, we investigated the roles of specific IN types in the recruitment of the GC population by stimulating the PP at various frequencies. Here, we delivered 10- and 30-Hz trains, which mimic inputs in the frequencies of θ and γ rhythm, respectively, from the EC, to the PP. We found that silencing of GAD65+ INs robustly enhanced all pSpikes during 10- and 30-Hz trains (–GAD65, green traces; 10 Hz, n = 9; 30 Hz, n = 11; Fig. 6a), whereas silencing of PV+ and SST+ INs differentially increased the pSpikes in the series (−PV, magenta traces; 10 Hz, n = 7; 30 Hz, n = 13; −SST, cyan traces; 10 Hz, n = 5; 30 Hz, n = 13; Fig. 6a). Overall, silencing of either GAD65+ or PV+ INs robustly enhanced the first pSpikes to the same extent in response to 10- and 30-Hz trains (Fig. 6b,c). However, silencing of GAD65+ INs resulted in a greater increase in the late phase of spike series at 30 Hz than in response to spike series at 10 Hz (Fig. 6b). Conversely, silencing of PV+ INs had a greater effect on late spike series at 10 Hz than that at 30 Hz (Fig. 6c). Furthermore, the effects on the later pSpikes upon silencing SST+ INs were similar during 10- and 30-Hz trains (Fig. 6d). To further examine how these INs differentially modulated a series of spikes in the GCs, we investigated the spiking probability of individual GCs in whole-cell recordings. We found that silencing of GAD65+ INs greatly increased the spiking probability of individual GCs during the entire series (–GAD65, n = 5; Fig. 6e,f), whereas inactivation of PV+ or SST+ INs differentially increased the spiking probabilities during series (−PV, n = 5; −SST, n = 8; Fig. 6e,f). Our results suggested that various types of INs differentially regulate GC responses to various activity patterns.

## Discussion

Spiking of dentate GCs in the rodent hippocampus is primarily regulated by synaptic inhibition originating from local GABAergic INs. Our results represent, to the best of our knowledge, the first study to provide causal evidence for the roles of specific types of dentate GABAergic INs in cortical input processing. We found that PV+ soma-targeting INs, not SST+ dendrite-targeting INs, contribute to feedforward inhibition in the DG in response to single entorhinal stimulation and thus regulate GC spiking. In addition, PV+ INs and SST+ INs differentially restrict GC spiking during θ and γ activities.

As noted above, our main finding strikingly contrasts with previous studies in the hippocampal CA1 region^38^,^50^. Using a pharmacogenetic approach, Lovett-Barron *et al*. found that silencing of SST+ dendrite-targeting INs, rather than silencing PV+ soma-targeting INs, increased the firing rate of CA1 PCs in response to Schaffer collateral input^50^. Furthermore, they found that PV+ INs primarily inhibit a subtype of SST+ INs named ‘bistratified INs’, which target the proximal dendrites of CA1 PCs during CA3 Schaffer collateral input. The disinhibition of SST+ bistratified INs can compensate for a withdrawal of perisomatic inhibition. With the existence of proposed reciprocal connections between SST+ INs and PV+ INs in the CA1 region, silencing of SST+ INs, in contrast, releases dendritic NMDA receptor-initiated electrogenesis, which cannot be overcome by disinhibition of PV+ INs, leading to increased firing of PCs. However, by using simultaneous dual recordings, Savanthrapadian *et al*. demonstrated that PV+ basket cells rarely target SST+ HIPP cells (2 of 80 dual recordings) in the DG, which suggests that functional connectivity from PV+ to SST+ INs has minor role in regulating network activity compared to that in CA1^45^. In addition to distinct inhibitory network mechanisms, GCs use different dendritic mechanisms for synaptic integration, which are also likely to account for this difference^19^. First, the GC dendrites are leaky and act as strong voltage attenuators for synaptic inputs^18^,^19^. Second, the GC dendrites are linear, meaning that they sum synaptic inputs linearly and are not designed for highly efficient synchrony detection^19^. Lastly, the GC dendrites lack dendritic spikes that would allow them to more efficiently bring EPSPs to action potential threshold^19^.

Recent work by Pouille *et al*. showed that feedforward GABAergic inhibition in CA1 expands the range of afferent input strengths that CA1 PC population can represent during single-shock stimulation^38^. Although the input threshold of spiking GCs was greatly reduced by gabazine (Fig. 2e), a large fraction of non-spiking GCs became spiking cells with a relatively high input threshold (open symbols in Fig. 2f). Similarly, optogenetic silencing of GAD65+ INs in the DG appeared to expand the range of input strength (Fig. 5b). As a result, GABAergic inhibition in the DG seems to restrict the input dynamic range of the GC population. Based on their relatively low threshold input strength and short spike delay, BC-like INs and ML-like INs (including molecular layer perforant pathway associated (MOPP) cells and neurogliaform cells (NGFCs)) are very likely to mediate this effect (Fig. 4e, Supplementary Fig. 2f–h). However, silencing of PV+ INs alone had only a slight effect on the input dynamic range of the GC population during single-shock PP stimulation. Taken together, we speculate that other non-PV+ INs may also contribute to feed-forward inhibition and play a role in the entorhinal-dentate inhibitory gate (Fig. 3f,g). Indeed, we found that MOPP cells and NGFCs fire earlier than GCs in response to the PP input and also exhibit relatively lower input strength (Supplementary Fig. 2d,e). In line with this idea, both cell types do provide dendritic GABAergic inputs to GCs^43^,^44^. Furthermore, single action potentials elicited in NGFCs can result in a slow GABA_A_ followed by a late GABA_B_ component in GCs^43^. Notably, although the amplitude of PV+ IN-evoked GABA_A_-mediated component is much greater than that evoked by NGFCs, the inhibitory charge transfer of the NGFC-evoked IPSCs is still comparable to that of PV+ IN-evoked events, underscoring the distinct and potentially important inhibitory role of the slow events evoked by NGFCs^43^. It is worth noting that Pouille *et al*. showed that feedforward GABAergic inhibition expands the dynamic range of the CA1 PC population by setting a global threshold for recruitment of CA1 PCs, which increases along with input strength^38^. Notably, in the DG, all soma-targeting INs and most ML-like INs, the major candidates involved in feedforward inhibition, are recruited by low input strengths (Fig. 4e, Supplementary Fig. 2). As a result, the number of these recruited INs in the DG may not increase further with stimulus strength.

Silencing of GAD65+ INs results in an increase in pSpikes, with effects of varying degrees with different timing during θ–γ activity, reflecting frequency-tuned distribution of inhibition in the DG. Indeed, our recent studies^22^,^37^ show that soma-targeting INs and dendrite-targeting INs are differentially recruited by excitatory inputs and in turn provide distinct spatiotemporal control over GC activity. Using paired recordings from INs and GCs, we found that inhibition in the DG is dominated by somatic GABAergic inputs during periods of sparse (0.2 Hz) presynaptic activity, whereas dendritic GABAergic inputs are rapidly shifted to powerful and sustained inhibition during periods of intense (30–90 Hz) presynaptic activity^22^. Together with the enhanced dendritic inhibition^22^, the delayed recruitment of SST+ inhibitory INs by repetitive PP stimulation at near-γ frequency may also contribute to marked increases in the later pSpikes while GAD65+ INs are inactivated (Fig. 6b). Notably, such frequency-tuned inhibition depends on the IN subtype. PV+ IN-mediated suppression at near-θ rhythm frequency is greater than that at γ-frequency (Fig. 6c). This is attributed to more reliable recruitment of PV+ INs and less depression of their output synapses at 10 Hz than at 30 Hz^20^,^25^. In contrast to PV+ INs, SST+ IN-mediated suppression shows no difference between 10 and 30 Hz (Fig. 6d). Intriguingly, the effect of GAD65+ IN-mediated suppression on the later pSpikes (3rd to 5th) at 30 Hz is much greater than the additive effect of silencing PV+ and SST+ INs at 30 Hz. This observation suggests that in addition to PV+ INs and SST+ INs, other types of INs in the DG are preferentially recruited during 30 Hz activity^43^,^44^.

In this study, the effect of gabazine application on pSpikes is more powerful than that of optogenetic silencing of GAD65+ INs. Incomplete silencing of GAD65+ INs, spontaneous release of GABA, and tonic extrasynaptic inhibition may account for the difference. Nevertheless, the increase in pSpikes after silencing of GAD65+ INs is still ~2-fold greater than that following silencing of PV+ INs, consistent with the idea that other types of GABAergic neurons may participate in the dentate gating. Notably, in addition to local INs, long-range-projecting GABAergic neurons in the EC and local INs are known to modulate DG excitability^55^. In the present study, monosynaptic inhibition mediated by long-range GABAergic neurons in the EC appears to be minimal under our recording conditions because PP evoked-IPSCs in GCs are disynaptic (Fig. 2d). It is consistent with the previous study^55^ showing that long-range GABAergic neurons in the EC preferentially target GABAergic INs in the DG. Nevertheless, we cannot completely exclude any possible effects resulted from optogenetic inactivation of long-range GABAergic neurons during either single shock or repetitive PP stimulation at near-θ rhythm frequency. On the other hand, it is known that local INs in the DG are highly heterogeneous^22^,^37^,^46^,^51^. Recordings from morphologically identified INs (Supplementary Fig. 2) show that both MOPP cells and NGFCs have lower threshold input strength than GCs and fire prior to GCs in response to PP stimulation. Interestingly, we found that some SST+ INs in the ML identified using *SST::Cre*;Ai14 mice were MOPP cells and NGFCs (unpublished data). In contrast, total molecular layer (TML) cells have greater threshold input strength than GCs (Supplementary Fig. 2f). Together with previous studies^43^,^44^, our results suggest that both MOPP cells and NGFCs may also participate in regulation of GC activation in response to cortical activity. The extensive nature of MOPP cell and NGFC axons within the ML also indicates that both cell types could broadly modulate the overall activity of the DG (also see ref. 43). It is worth mentioning that although NGFCs display slower and prolonged synaptic release^43^, they generate comparable inhibitory charge transfer and may play major roles in dentate gating during repetitive PP activation. Furthermore, our recent study^37^ revealed that although ML-like INs could only be recruited during the initial phase of 10 Hz trains by optogenetic activation of the medial PP, they could be reliably activated following the entire train stimulation of the hilar commissural/associational pathway. Hilar cells including mossy cells are known to have lower thresholds for synaptic activation by the PP than do GCs^56^. These results indicate that ML-like INs not only serve as feedforward inhibitors but also play an important role in feedback inhibition in the DG. Unfortunately, the function of MOPP cells and NGFCs is likely masked by HIPP-like cells, a large fraction of SST+ cells and cannot be addressed in this study. Thus, identification of cell-type-specific markers for MOPP cells and NGFCs and further development of cell-specific targeting of GABAergic INs will be useful for elucidation of their functional roles in hippocampal network function^57^.

Some potential caveats should be noted when we address the role of a genetically defined IN type in the network function using the optogenetic perturbation approach. First, we need to admit that total suppression of activity of specific IN classes is unlikely to occur in a healthy brain. Thus, the pronounced increase in the pSpike following silencing of PV+ INs may not be physiologically relevant. However, in terms of dissecting the causal role of specific IN classes in network function, optogenetic silencing of a defined IN type remains superior to pharmacological approaches. Second, tonic inactivation of specific IN classes in our study is less powerful to dissect out the precise temporal influence of the different classes of INs on the activity of postsynaptic cells. Third, a potential disinhibitory effect may contribute to the changes in pSpikes while we optogenetically inactivated PV+ or SST+ INs, particularly during prolonged inactivation. Accumulating evidence demonstrates that PV+ and SST+ INs also innervate other GABAergic INs, of either the same or different subtypes, thereby mediating disinhibitory control in the DG^22^,^45^,^58^,^59^,^60^. For example, in addition to the GCs, PV+ fast-spiking INs also innervate other PV+ INs and hilar commissural associational path (HICAP) cells, but rarely target HIPP cells in the DG^22^,^45^,^58^. All these synapses exhibit fast kinetics, large amplitudes, and multiple-pulse depression at γ-frequency activities^22^,^45^,^58^. Thus, the HICAP-mediated inhibitory effect on GC activity is likely to increase following optogenetic silencing of PV+ INs during single or repetitive cortical stimulation. On the other hand, SST+ HIPP cells have been shown to inhibit both PV+ BCs and HIPP cells, but they rarely target HICAP cells^22^,^45^. Although the IPSC amplitudes of HIPP-HIPP and HIPP-BC synapses are not as large as those at BC-HICAP synapses, all these synapse types show multiple-pulse facilitation at γ frequency^22^,^45^. Additionally, channelrhodopsin 2-mediated excitation of SST+ terminals demonstrates that they effectively control the activity of target INs^45^. Finally, connections might exist between HIPP cells and ML-like INs because the distribution of axons from HIPP cells largely overlaps with that of dendrites of ML-like INs. Thus, optogenetic silencing of SST+ INs during repetitive PP stimulation may enhance the inhibitory control of PV+ INs and ML-like INs over GC activity in the later stage of spike series, resulting in a small change in the pSpike (Fig. 6d). This compensatory effect should become more obvious at γ frequency because HIPP cell-mediated inhibition/disinhibition is much stronger during γ-frequency stimulation^22^,^45^. Therefore, better understanding the fine-tuned inhibitory networks in the brain requires further investigation into IN-IN interactions.

## Methods

### Electrophysiology

Transverse ventral hippocampal slices (400 μm) were prepared from the male Sprague-Dawley rats (at 3–4 weeks) or C57BL/6 mice (at 3–6 months). For optogenetic experiments, transgenic hemizygotic mice (at 2–4 months) of either sex were used. *Gad65-cre*, *Pvalb-cre*, and *Sst-cre* mice were obtained from the Jackson Laboratory (stock #010802, 008069, 013044, respectively; Bar Harbor, ME, USA) and maintained on a C57BL/6J background. Slices were sectioned in carbogen (95% O_2_ and 5% CO_2_)-bubbled ice-cold artificial cerebrospinal fluid (ACSF) containing the following (in mM): 125 NaCl, 25 NaHCO_3_, 1.25 NaH_2_PO_4_, 2.5 KCl, 25 glucose, 2 CaCl_2_, and 1 MgCl_2_, and they were then allowed to recover (25 min, 34 °C) in modified oxygenated ACSF containing the following (in mM): 87 NaCl, 25 NaHCO_3_, 2.5 KCl, 10 glucose, 75 sucrose, 0.5 CaCl_2_, and 7 MgCl_2_ before being transferred to standard oxygenated ACSF. Slices were placed in a submerged chamber and perfused with oxygenated ACSF (32 ± 1 °C) during experiments. The perfusion rate was 3.5–4.5 ml/min in all experiments. Animals were killed by decapitation in accordance with national and institutional guidelines and all experiments were conducted in accordance with methods approved by the Animal Care and Use Committee of National Yang-Ming University.

Neurons were visualized using infrared Dodt gradient contrast microscopy. Cell-attached and whole-cell recordings were performed with patch pipettes (8–10 MΩ) filled with internal solution containing the following (in mM): 146.5 K-gluconate, 3.5 KCl, 1.5 MgCl_2_, 5 HEPES buffer, 1.1 EGTA, 2 Na_2_ATP, and 10 phosphocreatine (pH = 7.3, 295–305 mOsm). All experiments were performed in the presence of the *N*-methyl-d-aspartate (NMDA) receptor antagonist D-2-amino-5-phosphonopentanoic acid (20 μM) and the GABA_B_ receptor antagonist CGP55845 (1 μM). Pharmacological blockade of NMDA receptors or GABA_B_ receptors is to prevent either excitatory or inhibitory synaptic plasticity mediated by these receptors under repetitive intensive stimulation during the experiments. The PP fibres were stimulated using a monopolar electrode placed in the subiculum to avoid direct activation of axons from DG INs, excluding the possibility that GABAergic conductance contributes to the fEPSP slope. Loose-patch and field recordings were performed with patch pipettes (6–10 MΩ, <1 MΩ) filled with ACSF. Signals were recorded with MultiClamp 700B amplifiers (Molecular Devices, Sunnyvale, CA, USA). Data were filtered at 2 kHz and sampled at 10 kHz with a Digidata 1440A interface (Molecular Devices) controlled by pCLAMP version 10.2 (Molecular Devices). For optogenetic silencing of eNpHR3.0-expressing neurons, amber light was emitted from a collimated light-emitting diode (590 nm) driven by a 4 channel LED Driver (Thorlabs, Newton, NJ, USA) under the control of a Digidata 1440A and Clampex version 10.2. Light was delivered through the reflected light fluorescence illuminator port and the ×63 objective. For the optogenetic experiments, light was applied 20 ms before each PP stimulation and lasted for 40 ms to cover the entire period of evoked EPSCs. Gabazine (1 μM) was used to block GABA_A_ receptors.

### Calibration of input strength

The input strength was calibrated as described previously^36^,^38^. The input strengths shown in this study were obtained from dual field electrode recordings with one placed in the ML, for fEPSPs, and another one in the GCL, for pSpikes. The pSpike was estimated based on underlying area (Temprana *et al*.^39^). The fEPSP slope evoked at any given stimulus intensity was normalized to the fEPSP slope elicited by a stimulus intensity that resulted in a pSpike at 95% of its maximum. A normalized fEPSP is referred to as the input strength. For each slice, the input strength was determined under control conditions. Notably, the fEPSP slope remained unchanged before and after gabazine treatment, indicating that the number of PP fibres stimulated either under control conditions or in the presence of gabazine, was the same for a given input strength within a slice. The input strength was calibrated only after the amplitude of the pSpike had remained stable for at least 10 min.

### Threshold stimulation and population activation curves

Neurons were recorded in the loose-patch or cell-attached configuration, and the PP was stimulated at different intensities to determine the threshold input strength defined as evoking a 50% spike probability in the recorded cells. Five to ten stimuli were tested at each intensity to calculate spiking probability. The 50% spiking probability of individual neurons was determined by fitting their spiking probability plotted against input strength with a sigmoid function *Y* = 100/[1 + 10^*p*(*x*_0_ − *x*)] as previously described^38^, where *x*_0_ is the input strength at 50% spiking probability, and *p* is the slope at *x*_0_. In a subset of the recordings, we determined threshold input strength in the whole-cell recording configuration. The threshold input strength did not differ notably among different recording configurations; therefore, results from all recordings were combined. The population activation curve is the cumulative distribution of the threshold input strengths of the neurons in that population.

### Virus injections

To selectively silence genetically defined INs in the DG, we used an AAV5 carrying a Cre-inducible eNpHR3.0-eYFP transgene (AAV5-EF1α-DIO-eNpHR3.0-eYFP) (for most experiments) or an AAV5 carrying Cre-inducible eNpHR3.0-mCherry (remaining experiments), both of which were produced by the University of North Carolina Vector Core Facilities, Chapel Hill, NC, USA. The virus injection procedure was performed as described previously^37^. Injection volume (0.5 μl at each location) and flow rate (0.1 μl/min) were controlled with a nanopump Controller (KD, Holliston, MA, USA). For targeting DG INs of the ventral hippocampus, coordinates from bregma were as follows: anteroposterior, −3.4 mm; mediolateral, ±2.8 mm; dorsoventral, −4.4 mm and −4.2 mm. Following suturing of the craniotomy, mice were returned to their home cages for recovery. All animals were allowed to recover for at least 6 weeks before the next experimental stage to allow gene transduction.

### Recovery of biocytin-filled neurons

Neurons were filled with biocytin (0.2–0.4%) during recordings. After ~30 min of recording, slices were fixed overnight with 4% paraformaldehyde in phosphate-buffered saline solution (0.1 M, pH 7.3). After washing with phosphate-buffered saline solution, slices were incubated with fluorescein isothiocyanate (FITC)-conjugated avidin-D (2 μl/ml; Invitrogen, Eugene, Oregon, USA) in phosphate-buffered saline solution containing 0.3% triton X-100 overnight at 4 °C. After washing, slices were embedded in Vectashield^®^ mounting medium (Vector Laboratories, Burlingame, CA, USA).

### Image acquisition and anatomical reconstruction

For 3-D reconstruction of biocytin-labelled cells, high-resolution two-photon images of neurons were acquired. Labelled neurons were examined using a two-photon microscope with a pulsed titanium: sapphire laser (Chameleon-Ultra II tuned to 800 nm; Coherent, Portland, OR, USA) attached to a Leica DM6000 CFS (Leica, Wetzlar, Germany) that was equipped with a 63×/0.9 numerical aperture (NA) water immersion objective (objective type HCX APO L). The cell morphology was reconstructed from a stack of 148–212 images per cell (voxel size, 758 nm in the *x*-*y* plane; 1 μm along the *z*-axis). Image stacks belonging to individual cells were imported into the Neuromantic 1.6.3 software for 3-D reconstruction^61^. We quantified the axonal density along the radial axis as previously described^22^. In brief, we counted the number of intersections made by the axons with lines running parallel to the border between the GCL and the ML and interspaced by 10 μm.

### Statistics

Values are expressed as the means ± the standard error of the mean (SEM). Error bars also indicate the SEM in the figures. Statistical significance was tested using the Wilcoxon signed-rank test for paired data or the Wilcoxon rank-sum test for unpaired data at the significance level (P) as indicated, using Prism version 5.0 (GraphPad Software, La Jolla, CA, USA). Two-way repeated-measures ANOVA was used to compare the effects of IN silencing on GC population activity at different input strengths or input frequencies.

## Additional Information

**How to cite this article**: Lee, C.-T. *et al*. Causal Evidence for the Role of Specific GABAergic Interneuron Types in Entorhinal Recruitment of Dentate Granule Cells. *Sci. Rep*. **6**, 36885; doi: 10.1038/srep36885 (2016).

**Publisher's note:** Springer Nature remains neutral with regard to jurisdictional claims in published maps and institutional affiliations.

## Supplementary Material

Supplementary Information
