# Supplementary figures and images for: Causal Evidence for the Role of Specific GABAergic Interneuron Types in Entorhinal Recruitment of Dentate Granule Cells
Source: Sci Rep. 2016 Nov 10;6:36885. doi: 10.1038/srep36885 (PMC5103275; doi:10.1038/srep36885)

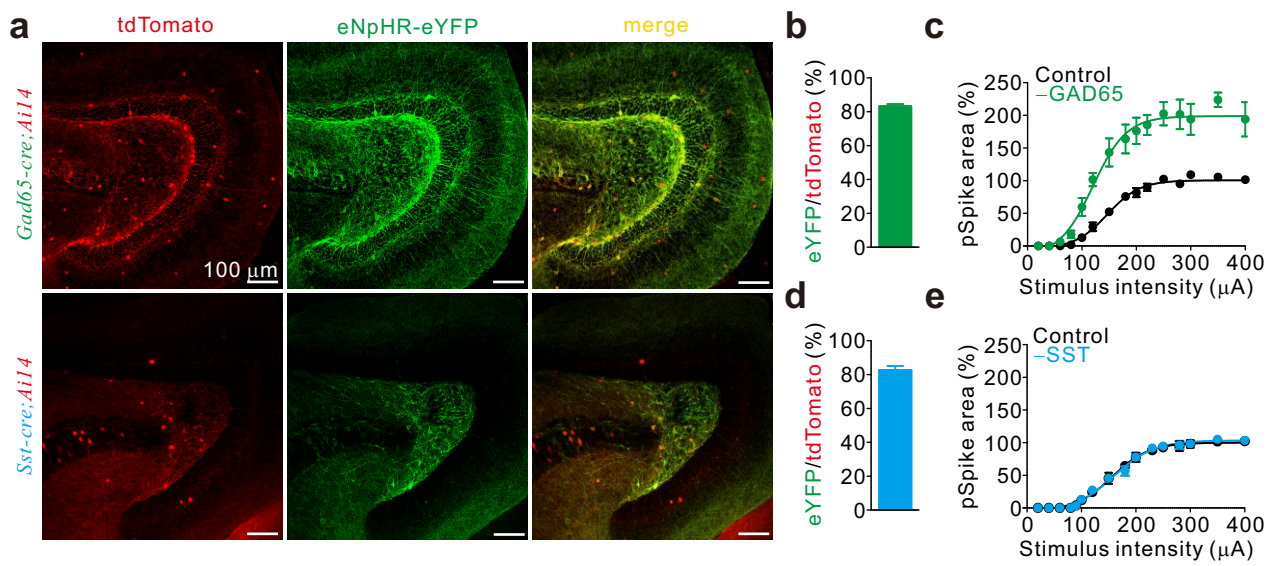

Fig.S1 Lee et al.

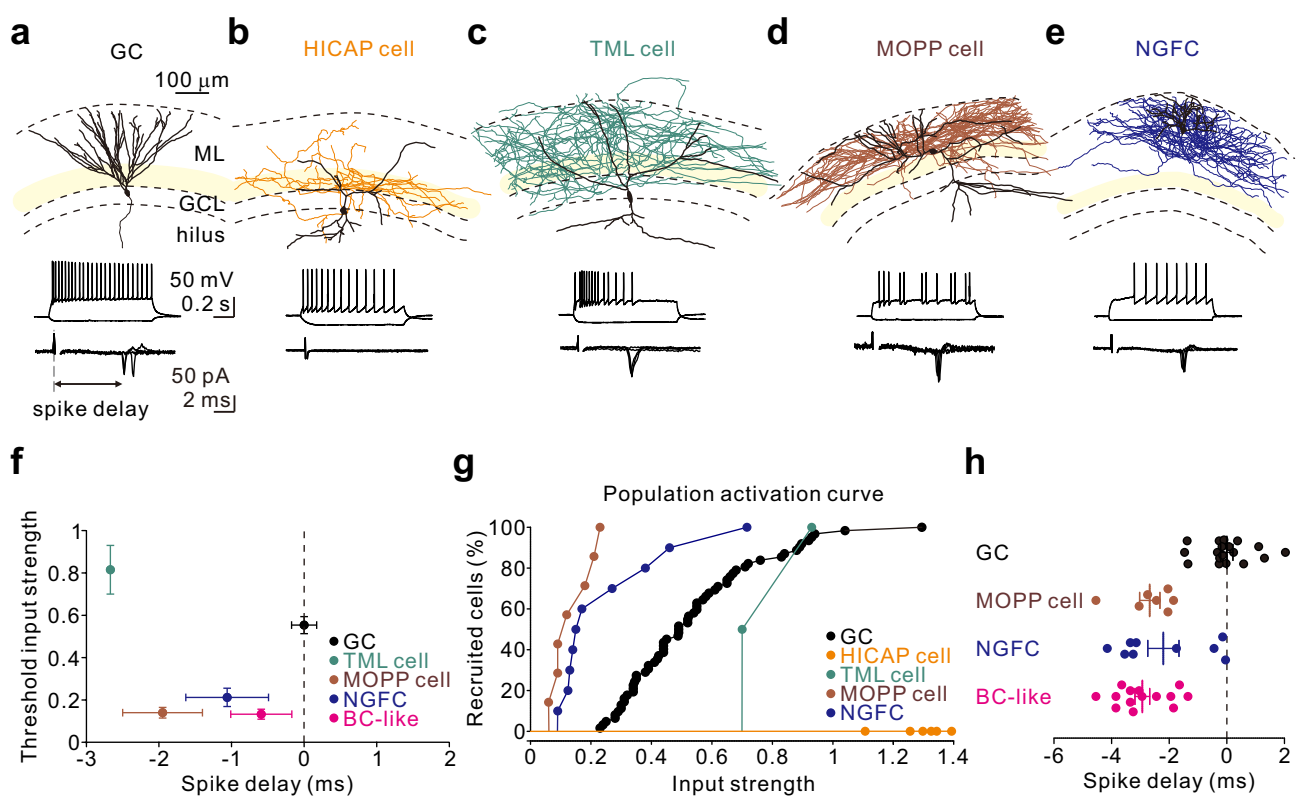

Fig.S2 Lee et al.

Supplement: Supplementary Information [file srep36885-s1.pdf]
